# Immobilization and Characterization of a Recombinant Thermostable Lipase (Pf2001) from *Pyrococcus furiosus* on Supports with Different Degrees of Hydrophobicity

**DOI:** 10.4061/2010/180418

**Published:** 2010-10-28

**Authors:** Roberta Vieira Branco, Melissa Limoeiro Estrada Gutarra, Denise Maria Guimarães Freire, Rodrigo Volcan Almeida

**Affiliations:** Departamento de Bioquímica, Instituto de Química, Universidade Federal do Rio de Janeiro, RJ 21941-909, Rio de Janeiro, Brazil

## Abstract

We studied the immobilization of a recombinant thermostable lipase (Pf2001Δ60) from the hyperthermophilic archaeon *Pyrococcus furiosus* on supports with different degrees of hydrophobicity: butyl Sepabeads and octadecyl Sepabeads. The enzyme was strongly adsorbed in both supports. When it was adsorbed on these supports, the enzyme showed 140 and 237% hyperactivation, respectively. The assessment of storage stability showed that the octadecyl Sepabeads immobilized enzyme showed 100% of residual activity after 30 days of storage. However, the greatest stability at 70°C was obtained in butyl Sepabeads immobilized enzyme, which retained 77% activity after 1 hour incubation. The maximum activity of the immobilized preparations was obtained with the pH between 6 and 7, at 70°C. Thus, this study achieved a new extremophilic biocatalyst with greater stability, for use in several biotechnological processes.

## 1. Introduction

Carboxylesterases (E.C.3.1.1.1) and lipases (E.C.3.1.1.3) are enzymes that are classified as hydrolases, which in aqueous media catalyze the hydrolysis of ester bonds, generating alcohol and carboxylic acids. These enzymes catalyze various reactions, which sometimes have high chemo- and enantioselectivity, which explains their use in several sectors, such as the food, paper, textile, and detergent industries, wastewater treatment, fine chemistry, and pharmaceutical synthesis [1–5]. A number of lipases present a polypeptide chain called “lid” covering the active site and may exist in two different forms: one of them, where the active site of the lipase is isolated from the reaction medium by lid (closed form); the other conformation, presenting the lid displaced and the active centre exposed to the reaction medium (open form). This conformational change from closed to open form causes an increasing in enzyme activity when lipases are exposed to insoluble substrates, called “interfacial activation” [6]. This structural phenomenon was used to differentiate lipases from esterases which do not show this activation. However, some studies showed that neither all lipases presented interfacial activation, nor lid, and interestingly some lipases showed interfacial activation only for specific substrates. So these characteristics are not sufficient to differentiate lipases from esterases. Although there are works with others approaches to differentiate these enzymes [7], the definition of lipases more accepted is lipases are carboxylesterases that catalyze the hydrolysis (and synthesis) of ester bonds with long chain fatty acids (C ≥ 10) [8]. This mechanism of action presented by some lipases has been used as a tool to develop new and simpler methods for lipase immobilization in which the interfacial activation could be used as a source of new techniques of lipase engineering via directed immobilization [9].

One of the main problems of these biocatalysts is their instability under adverse conditions (e.g., organic solutions, extremes of temperature, ionic strength, pH, and pressure) which often makes the involved processes economically unfeasible. In some cases, the stability of an enzyme can be improved by immobilization. The use of immobilized enzymes instead of soluble enzymes also presents other advantages for industrial processes: ease of biocatalyst and product recovery; continuous processing; prevention of aggregate formation in organic media; reduction of denaturant effects; and modification of physical-chemical properties. However, the immobilization process must be well designed. Random immobilizations may not improve the rigidity of the enzyme, and in some cases the stability of the enzyme may decrease after immobilization, for example, if the support has undesirable interactions with the enzyme [10].

Aside from the use of immobilized enzymes, the other alternative investigated here to overcome the problem of biocatalyst stability is the use of enzymes from extremophilic organisms, in view of their natural ability to withstand extreme conditions [11–13]. The difference in thermostability between mesophilic and thermophilic proteins is due to the smaller tendency that thermophilic enzymes have to unfold. This is because these thermoenzymes have a higher number of interactions (e.g., hydrogen bonding, hydrophobic interactions, and disulfide bonds) than the mesophilic enzymes. In addition, thermophilic enzymes have a conformational structure, more rigid and packed, and presented low activity at low and moderate temperatures, which is a trouble because many interesting compounds in fine chemistry are stable only at these temperatures [14]. This kind of problem has been overcome to thermophilic lipases using immobilization on hydrophobic supports [15]. So the immobilization techniques in addition to use of extremophilic enzymes could amplify the enzymes application field and feasibility of some biotechnological processes [16–19]. 

Lipases and esterases have been immobilized by different processes, such as occlusion, adsorption by covalent and ionic bonds, and physical adsorption [20–24]. In the last case, the use of hydrophobic supports is most promising, because these supports mimic the enzymes' natural medium and can often promote hyperactivation, highly selective adsorption, purification, increased enantioselectivity, and strong but reversible immobilization, allowing the support being reused after the enzyme has been deactivated [25–27]. Some researchers have also been working with thermophilic enzymes immobilized on hydrophobic supports. For example, [28] who immobilized a lipase from the thermophil *Geobacillus thermoleovorans* on a support of polypropylene with micropores, and observed that it increased its thermostability after the immobilization process, with residual activity even after 1 hour of incubation at 100°C. Nawani et al. [29] studied a lipase from *Bacillus* sp. This enzyme showed optimal activity at 60°C and optimum pH 8.5. This lipase was immobilized on silica and on HP20. These immobilized biocatalysts showed pHs very close to the soluble enzyme, and the temperature increased by 5°C. They also showed high thermostability with a half life 2.5 times that of the soluble enzyme. Palomo et al. [30] immobilized a lipase from *Bacillus thermocatenulatus* (BTL2) in various supports with different characteristics, and observed that BTL2 immobilized on octadecyl Sepabeads showed hyperactivation and, compared with other immobilized preparations, showed the highest values of stability at a temperature of 65°C, and in 30% dioxane, retained virtually 100% of its activity in both experiments. 

Almeida et al. [31] identified an enzyme from the extremophilic species *Pyrococcus furiosus*, which they cloned and expressed in *Escherichia coli*. This enzyme showed higher activity for 4-methylumbelliferyl-heptanoate being first classified as an esterase. The optimal temperature and pH for this enzyme were found to be 60°C and 7.0, respectively. In this work the authors constructed a structural model by homology modeling. They observed a putative catalytic triad (Ser149, Asp233 and His264) and no lid domain.

In this study, we investigated the immobilization and characterization of this enzyme from *P. furiosus* on commercial supports with different degrees of hydrophobicity. So this study makes a contribution to the literature investigating two simultaneous biotechnological alternatives for obtaining a highly stable biocatalyst: the use of an enzyme from an extremophilic organism and its immobilization by adsorption on hydrophobic supports. 

## 2. Materials and Methods

### 2.1. Materials

The 4-Methylumbelliferyl-heptanoate (Muf-Hept) and gum arabic were acquired from Sigma (Sigma Chemicals, USA). The octadecyl Sepabeads (OS) and butyl Sepabeads (BS) were purchased from Mitsubishi Chemical Corporation. All other chemicals were of the highest reagent grade commercially available. 

### 2.2. Expression of the Recombinant Enzyme Pf2001Δ60

The Pf2001Δ60 enzyme was produced as described by Almeida et al. [31], but with some alterations. *E. coli* BL21 (DE3) pLysS bearing the Pf2001Δ60 gene was grown in LB broth (0.5% yeast extract, 1.0% tryptone, and 0.5% NaCl) containing ampicillin (100 *μ*g·L^−1^) and chloramphenicol (12.5 *μ*g·L^−1^), at 35°C and 200 rpm, until OD_600 nm_ 0.3 was reached. The enzyme was then induced with the addition of 0.5 mM IPTG and further incubation for 3 hours. The cells were centrifuged and stored at −20°C until they were used. The enzyme extract was obtained by resuspending the frozen cells in sodium phosphate buffer (50 mM, pH 7.0) and then disrupting them by sonication (until the crude extract was observed to be homogeneous). The crude extract was centrifuged (11,000 g at 4°C for 5 minutes) and the supernatant was used in the enzyme activity assay.

### 2.3. Activity Assay: Soluble Enzyme

Enzyme activity was measured according to the method described by Prim et al. [32] using Muf-Hept as substrate and a Cary Eclipse Fluorescence Spectrophotometer from Varian. 0.6 mL emulsion was used containing 0.1% gum arabic in sodium phosphate buffer (50 mM, pH 7.0), and 2.4 *μ*L Muf-Hept stock solution (25 mM in ethylene glycol monomethyl ether) to which 60 *μ*L enzyme extract was added. The enzyme activity was determined “on line” at 70°C by measuring the rise in fluorescence emissions (*λ*_ex_ = 323 nm and *λ*_em_ = 448 nm) during the reaction period. The reactions were carried out at the initial rate used. One unit of enzyme activity was defined as the amount of enzyme required to release 1 *μ*mol Muf per minute under assay conditions. The standard curve was generated using Muf.

### 2.4. Activity Assay: Immobilized Enzyme

The immobilized enzyme activity was determined according to Almeida et al. [33] with minor modifications. 7 mg immobilized enzyme was added to 10 mL reaction mixture (0.1% gum arabic in a 50 mM sodium phosphate buffer at pH 7.0) in a batch-stirred tank reactor with magnetic stirring (200 rpm) at 70°C. The reaction mixture was incubated until 70°C, when the immobilized biocatalyst was added, followed by 40 *μ*L Muf-Hept (25 mM in ethylene glycol monomethyl ether) to start the reaction. The progress of the reaction was evaluated as described in the previous section, and the fluorescence was measured after 30 s, 2 minutes, 3 minutes, and 4 minutes. The total protein concentration was established by the Bradford's method, using bovine serum albumin as standard [34].

### 2.5. Enzyme Immobilization

The immobilization process was carried out by adding 33 mL enzyme solution (protein concentration = 0.545 mg·mL^−1^ in sodium phosphate buffer 50 mM, pH 7.0) to 1.0 g of the support at 5°C with magnetic stirring to aid the adsorption process. After 2 hours the immobilized enzyme was rinsed with sodium phosphate buffer (50 mM, pH 7.0) to remove the nonadsorbed enzyme. Next, vacuum filtration was carried out and the immobilized preparation was placed in a desiccator for two days until the obtainment of a constant weight. The kinetics of the immobilization process were evaluated by taking samples of the supernatant at different intervals (0–120 minutes) to make a total protein analysis by the Bradford method and enzyme activity at 70°C using Muf-Hept as a substrate [32].

The immobilization procedure, using different enzyme concentrations for each support, was performed by adding 1.0 mL enzyme solution (0.3 to 9.0 mg·mL^−1^ protein) to butyl or octadecyl Sepabeads. Total protein [34] and activity [32] were analyzed in the supernatant, and the immobilized biocatalyst activity was also evaluated.

The immobilization parameters used to evaluate the enzyme immobilization process were calculated as described in the following equations:

Immobilization efficiency (*E*%):

(1) $$\begin{array}{cc} E\left(\text{\%}\right) & =\frac{U_{A}-U_{E}}{U_{A}}\cdot 100. \end{array}$$

Retention activity parameter (*R*%):

(2) $$\begin{array}{cc} R\left(\text{\%}\right) & =\frac{U_{H}}{U_{A}-U_{E}}\cdot 100, \end{array}$$

where *U*_*H*_ is the units of immobilized enzyme; *U*_*A*_ is the added units or units of activity offered for immobilization; *U*_*E*_ is the output units or units of activity in the solution after immobilization procedure.

Protein yield (*η*%):

(3) $$\begin{array}{cc} \eta \left(\text{\%}\right) & =\frac{N_{A}-N_{E}}{N_{A}}\cdot 100, \end{array}$$

where *N*_*A*_ is the protein offered for immobilization; *N*_*E*_ is the output protein in the solution after immobilization.

### 2.6. Characterization of the Immobilized Biocatalysts

The characterization of the immobilized biocatalysts was carried out using a factorial design (3^2^), with replicates at the central point. Two variables were studied: the pH (6, 7 and 8) and the temperature (50°, 70° and 90°). Statistical analyses of the results were performed using Statistica v6.0 software. A model was developed to describe the enzyme activity achieved as a function of the two variables under analysis. The statistical parameters used to corroborate these variables were the *t*-test and the *P* value. Only the statistically (*P* < .05) and marginally significant terms were included in the models.

### 2.7. Thermal and Storage Stability

The storage stability of the immobilized biocatalysts was tested over a period of 50 days at room temperature. Their thermal stability was tested by incubating 7 mg immobilized enzyme in 1 mL reaction mixture (0.1% gum arabic in 50 mM sodium phosphate buffer, pH 7.0) at 70°C, and the activity was measured after this incubation period, as described in 2.4. The effect of Triton X-100 on the stability of the preparations was studied by adding 0.4% v/v Triton X-100 to the reaction mixture.

## 3. Results and Discussion

### 3.1. Immobilization Time Course

**Figure 1:**
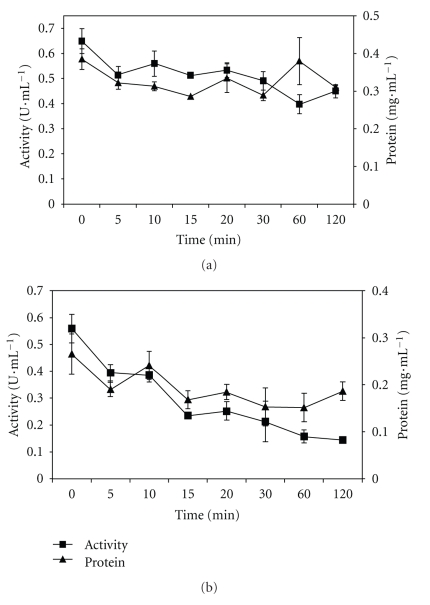
Immobilization time course of recombinant enzyme from *P. furiosus* immobilized on (a) butyl and (b) octadecyl Sepabeads in terms of activity (U·mL^−1^) and protein concentration (mg·mL^−1^). The standard deviations are indicated in the figure.

The measurements of activity and total protein during immobilization on butyl Sepabeads and octadecyl Sepabeads supports are shown in Figure 1. The immobilization efficiency (*E*%) and protein yield (*η*%) of the enzyme immobilized on different supports are shown in Table 1.

After two hours' immobilization of the *P. furiosus* enzyme on butyl Sepabeads (Figure 1(a) and Table 1), 20% and 31% of the initial protein and activity had been adsorbed, respectively. In the immobilization on octadecyl Sepabeads, 30% of protein and 74% of activity were adsorbed (Figure 1(b) and Table 1). The immobilization adsorption rate, in terms of activity (compared with total protein) was greater on octadecyl Sepabeads (Figure 1(b)) than on butyl Sepabeads (Figure 1(a)). Esterases and lipases have been immobilized on hydrophobic supports in several works [9, 15, 16, 18, 33, 35–37]. The use of hydrophobic supports is most promising because these supports mimic the enzymes' natural medium and can often promote hyperactivation, highly selective adsorption, purification, increased enantioselectivity, and strong but reversible immobilization, allowing the support to be reused after the enzyme has been deactivated [25–27]. The results of the immobilized enzyme on octadecyl Sepabeads and butyl Sepabeads indicate that the more hydrophobic support showed a greater difference between total adsorbed protein and enzyme activity. When support hydrophobicity is increased unspecific adsorption will be raised, adsorbing a higher total protein content. This phenomenon was observed in our results. Furthermore, we observed that higher activity content was adsorbed on octadecyl Sepabeads.

Adsorption at low ionic strength on hydrophobic supports seems to be capable of preferentially absorbing lipases and esterases, rather than the other proteins present in the crude extract. Bastida et al. [38] immobilized a lipase from *H. lanuginosa* on octyl agarose and observed that the activity of the supernatant decreased rapidly: about 50% of the activity was immobilized in 30 minutes, indicating fast enzyme immobilization. Wilson et al. [39] immobilized a lipase from *Alcaligenes* sp. (lipase QL) on octadecyl Sepabeads, and observed that 100% of this enzyme was adsorbed in one hour. Segura et al. [40] immobilized an extract containing lipase from pig pancreas on octyl agarose, and achieved 70% adsorbed activity in only one hour of immobilization. These results are in agreement with the results of this study, especially with regard to octadecyl Sepabeads.

### 3.2. Characterization of Immobilized Biocatalysts

The effect of temperature and pH on the enzyme activity of *P. furiosus *immobilized on butyl Sepabeads and octadecyl Sepabeads was investigated using a factorial design (3^2^). 

**Figure 2:**
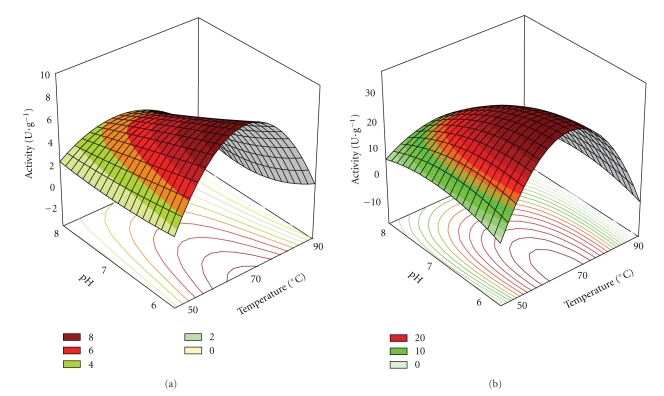
Response surface for immobilized enzyme activity on (a) butyl and (b) octadecyl Sepabeads (U·g^−1^ of support) as a function of temperature and pH. The surfaces were constructed only with the statistically significant variable.

The effects and regression coefficients for the studied variables were calculated for the immobilized enzymes on butyl Sepabeads and octadecyl Sepabeads, in linear and quadratic terms of the second-order model. The pure errors of both biocatalysts were low, indicating that the experiments had a good degree of reproducibility. The analysis also showed high determination coefficients (*R* ^2^), which demonstrates that the model described the system satisfactorily. Thus, coded models for immobilized biocatalysts on butyl Sepabeads (1) and octadecyl Sepabeads (2) were proposed, and each model generated a surface response, as shown in Figure 2.

(1) *A* = 3.22721 − 1.17333. *T* + 2.12081. *T* ^2^ − 1.26167. pH − 0.21000. *T*. pH − 0.20000. *T*. pH^2^ − 0.76750. *T* ^2^. pH + 0.56747. *T* ^2^. pH2. *R* ^2^ = 0.9968.

(2) *A* = 12.27460 − 4.125. pH + 1.4694. pH^2^ − 3.97. *T* + 7.5269. *T* ^2^ − 3.01875. pH. *T* ^2^ *R* ^2^ = 0.9713.

Analyzing Figure 2, it can be observed that the optimal temperature for the immobilized enzyme on both supports was 70°C, while the optimal pH was between 6 and 7.

### 3.3. Thermal and Storage Stability

The immobilized enzyme on butyl Sepabeads maintained 60% of its initial activity during 50 days of storage, while the immobilized biocatalyst on octadecyl Sepabeads maintained about 100% of its stability during the same storage period. These results indicate that the more hydrophobic the support, the greater the stability of the biocatalyst. A possible explanation for this behavior is that the more hydrophobic a support, the less water it will retain, and therefore all the deactivation processes that are related to the hydration percentage will be less likely, ensuring better preservation of the enzyme structure. 

**Figure 3:**
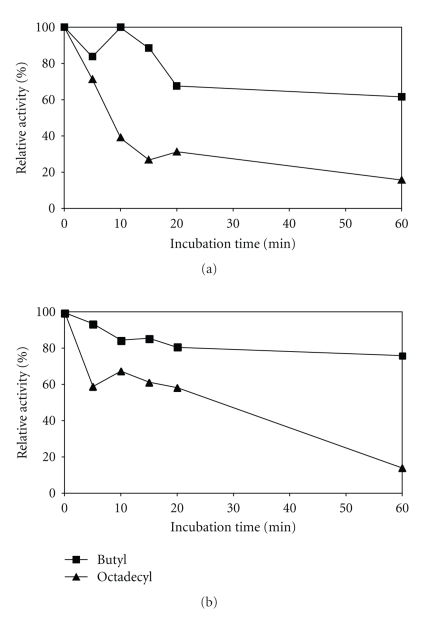
Thermal stability at 70°C of the enzyme immobilized on butyl and octadecyl Sepabeads. (a) Without Triton X-100 and (b) with 0.4% Triton X-100.

The thermal stability of the immobilized enzyme on different supports was evaluated with and without Triton X-100 (polyoxyethylene octyl phenyl ether), a compound that has been used as an emulsifier in reactions catalyzed by lipases and/or esterases [41]. The stability of the enzyme immobilized on butyl Sepabeads and octadecyl Sepabeads at 70°C for 1 hour incubation in the presence or absence of Triton X-100 is shown in Figure 3.  Table 2 presents the results of the initial and residual activity of immobilized biocatalysts after 1 hour incubation at 70°C.

**Figure 4:**
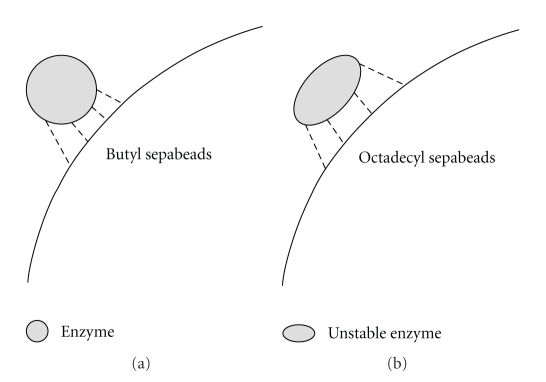
Hypothesis to explain the hydrophobic effect of the support on the biocatalysts' thermal stability: (a) weaker interactions between support and enzyme assure the integrity of the biocatalyst structure, while (b) stronger interactions deform and destabilize the structure.

Thermal stability was reduced as the hydrophobicity of the supports rose, with and without Triton X-100 (Figure 3). Such fact could be explained by the hypothesis illustrated in Figure 4. Octadecyl Sepabeads—the support with higher hydrophobicity—probably had stronger interaction with the enzyme than the butyl Sepabeads, and consequently caused its destabilization at a high temperature (Figures 3 and 4). The enzymes from extremophiles such as *P. furiosus* are naturally more rigid than those from mesophilic microorganisms, and immobilizing them in extremely hydrophobic supports could contribute to destabilizing their structure, damaging the thermal stability of the enzyme. This hypothesis could explain the lower optimal temperature found for the immobilized enzyme compared with the soluble enzyme observed by Alquéres (unpublished).

The effect of Triton X-100 on biocatalyst stability, shown in Figure 3 and Table 2, suggests that this detergent has the effect of protecting the enzyme, increasing its stability in both butyl Sepabeads and octadecyl Sepabeads. This protective effect was observed for octadecyl Sepabeads during the initial incubation period (20 minutes), which confirms the hypothesis that the enzyme structure was more affected by its interaction with this adsorbent, reducing the protective effect of the detergent. It is important to emphasize that no enzyme desorption was observed when Triton X-100 was used (data not shown) suggesting a strong adsorption.

Table 2 shows that the initial activity levels were higher in the presence of Triton X-100. As an emulsifier, Triton X-100 can modify the aggregation of the substrate or the structure of the enzyme, or raise the surface of the interface, leading to greater substrate availability and the higher initial activity of the immobilized biocatalyst [41, 42].

According to Wilson et al. [42] a lipase from *Alcaligenes* sp. (soluble and immobilized) incubated in emulsion without Triton X-100 at 70°C for 9 hours maintained 50% of its initial activity. However, when Triton X-100 was added, the enzyme lost almost 100% of its initial activity in 90 min of incubation. This loss of activity is related to the disaggregation of the native bimolecular form of this enzyme, induced by Triton X-100. The stability of this lipase from *Alcaligenes *sp. was diminished when it was incubated with Triton X-100, unlike the enzyme from *P. furiosus.* These results demonstrate that Triton X-100 can be harmful or harmless to enzymes, depending on their structure and how Triton X-100 affects them. 

In the studies by Almeida et al. [31] and Alquéres (unpublished) of crude and purified preparations, respectively, the soluble enzyme was found to be stable at 70°C in the presence of Triton X-100. Their results are compatible with the results found in this study, where the enzyme immobilized in butyl Sepabeads remained stable at up to 70°C in the presence of Triton.

### 3.4. Effect of Enzyme Concentration on Retention Activity

The results of the experiments to immobilize the enzyme from *P. furiosus *on butyl Sepabeads and octadecyl Sepabeads supports show that the lower the protein concentration, the higher the retention activity achieved for all the tested supports. For the enzyme immobilized on butyl Sepabeads hyperactivation of almost 140% occurred at 0.3 mg·g^−1^ of initial protein per mass of support, while for the enzyme immobilized on octadecyl Sepabeads, there was hyperactivation of approximately 237% at 0.8 mg·g^−1^. In other words, hyperactivation was observed only when lower protein concentrations were used in the immobilization process. In this situation, although the highest protein/support ratio was still far short of the saturation concentration of the supports, it would appear that more suitable microenvironments (enzyme, proteins, support, and substrate) are formed when a lower amount of protein is immobilized on the support. It may be that a greater concentration of enzyme/protein used in the immobilization process could generate a phenomenon known as “overcrowding”, that is, the enzyme and protein could be adsorbed on each other, preventing the exposure of the active site to catalysis. This gives rise to a considerable reduction in retention activity, and hardly any hyperactivation is seen. 

Almeida et al. [33] established the retention activity in the immobilization of the esterase from *P. furiosus* at different protein/support ratios. They observed that greater hyperactivation occurred at an intermediate protein/support ratio of 18 mg protein^−1^ gram of Accurel MP1000, for which hyperactivation reached 340%. To explain this, the authors suggested that a microenvironment was formed, involving the support surface, the active site of the enzyme and the substrate, which favored the catalytic efficiency of the enzyme. This is corroborated by the fact that hyperactivation is dependent on the protein/support ratio used in the immobilization process. 

Palomo et al. [43] used octadecyl Sepabeads to immobilize lipases from *Candida antarctica, Mucor miehei, *and* Candida rugosa *by interfacial adsorption. The lipase from *Mucor miehei *showed fivefold hyperactivation when immobilized on octadecyl Sepabeads, due to the stabilization of the open form of the lipase when it is adsorbed by an extremely hydrophobic support. Other researchers have observed hyperactivation when enzymes are immobilized on hydrophobic supports. Wilson et al. [42] observed 35% hyperactivation when a lipase from *Alcaligenes* sp. was immobilized on octadecyl Sepabeads. Bastida et al. [38] immobilized a lipase from *H. lanuginosa *on octyl agarose and observed 20-fold hyperactivation. Segura et al. [40] immobilized an extract containing lipases from pig pancreas on octyl agarose and observed hyperactivation of nearly 175%. This phenomenon was due to the high hydrophobicity of the support, in other words, the existence of large hydrophobic areas in the octyl agarose, where the lipases would simultaneously be adsorbed and have interfacial activation.

## 4. Conclusions

This study achieved a new biocatalysis of an extremophilic organism being strongly adsorbed on hydrophobic supports, with great thermal and storage stability, for use in biotechnological processes that require such characteristics.

The immobilization of a recombinant enzyme from *P. furiosus* (Pf2001Δ60) by adsorption on supports of commercial origins with different degrees of hydrophobicity allows the hyperactivation of this enzyme (140% for butyl Sepabeads and 237% for octadecyl Sepabeads) and the obtainment of biocatalysts with attractive thermostability characteristics.

Although its immobilization on supports with different degrees of hydrophobicity caused the enzyme to have different hyperactivation patterns, the temperature and pH (*T* = 70°C and pH = 6-7) remained close to their optimal levels for biocatalytic action.

In previous work Almeida et al. (2006) [31] cloned and expressed the gene PF2001 from *P. furiosus* in *E. coli*, characterizing the enzyme as an esterase according its substrate preference to MUF-Hept (C < 10). This enzyme was immobilized on microporous polypropylene at low ionic strength, showing the hyperactivation phenomenon [33]. In this work the same enzyme was immobilized on more hydrophobic supports showing, once more, the hyperactivation phenomenon. The immobilization on hydrophobic supports under low ionic strength and hyperactivation is a characteristic of lipases suggesting that Pf2001 enzyme is a lipase not an esterase.

## Figures and Tables

**Table 1:** Immobilization efficiency (*E*%) and protein yield (*η*%) of immobilized recombinant enzyme from *P. furiosus* on butyl Sepabeads and octadecyl Sepabeads.

Support	*E*(%)	*η*(%)
butyl Sepabeads	31	20
octadecyl Sepabeads	74	30

**Table 2:** Thermal stability at 70°C for one h of incubation in the presence (+) or absence (−) of Triton X-100. BS: butyl Sepabeads; OS: octadecyl Sepabeads.

Support	Initial activity (U/g)		Residual activity (%)	
	+	−	+	−
butyl Sepabeads	3.02	1.78	77	61
octadecyl Sepabeads	6.7	2.43	16	15.6

## Symbols and Abbreviations

Muf-Hept: 4-Methylumbelliferyl-heptanoate
Muf: Methylumbelliferil
OS: Octadecyl sepabeads
BS: Butyl sepabeads
IPTG: Isopropyl-*β*-D-thiogalactopyranoside
*P. furiosus:*: *Pyrococcus furiosus*
BSA: Bovine Serum Albumin
*E*(%): Immobilization efficiency
*R*(%): Retention activity parameter
**η**(%): Protein yield
*U*_*H*_: Units of immobilized enzyme
*U*_*A*_: Added units, or units of activity offered for immobilization
*U*_*E*_: Output units, or units of activity in the solution after immobilization procedure
*N*_*A*_: Protein offered for immobilization
*N*_*E*_: Output protein in the solution after immobilization
*T*: Temperature.
